# Antipsychotic plasma levels in the assessment of poor treatment response in schizophrenia

**DOI:** 10.1111/acps.12825

**Published:** 2017-10-26

**Authors:** R. McCutcheon, K. Beck, E. D'Ambrosio, J. Donocik, C. Gobjila, S. Jauhar, S. Kaar, T. Pillinger, T. Reis Marques, M. Rogdaki, O. D. Howes

**Affiliations:** ^1^ Department of Psychosis Studies Institute of Psychiatry, Psychology & Neuroscience King's College London London UK; ^2^ MRC London Institute of Medical Sciences Hammersmith Hospital London UK; ^3^ Faculty of Medicine Institute of Clinical Sciences Imperial College London London UK; ^4^ South London and Maudsley NHS Foundation Trust London UK

**Keywords:** adherence, compliance, therapeutic drug monitoring, treatment‐resistant, psychosis

## Abstract

**Objective:**

Treatment resistance is a challenge for the management of schizophrenia. It is not always clear whether inadequate response is secondary to medication ineffectiveness, as opposed to medication underexposure due to non‐adherence or pharmacokinetic factors. We investigated the prevalence of subtherapeutic antipsychotic plasma levels in patients identified as treatment‐resistant by their treating clinician.

**Method:**

Between January 2012 and April 2017, antipsychotic plasma levels were measured in 99 individuals provisionally diagnosed with treatment‐resistant schizophrenia by their treating clinicians, but not prescribed clozapine. Patients were followed up to determine whether they were subsequently admitted to hospital.

**Results:**

Thirty‐five per cent of plasma levels were subtherapeutic, and of these, 34% were undetectable. Black ethnicity (*P* = 0.006) and lower dose (*P* < 0.001) were significantly associated with subtherapeutic/undetectable plasma levels. Individuals with subtherapeutic/undetectable levels were significantly more likely to be admitted to hospital (*P* = 0.02).

**Conclusion:**

A significant proportion of patients considered treatment‐resistant have subtherapeutic antipsychotic plasma levels, and this is associated with subsequent admission. The presence of subtherapeutic plasma levels may suggest a need to address adherence or pharmacokinetic factors as opposed to commencing clozapine treatment. While antipsychotic levels are not recommended for the routine adjustment of dosing, they may assist with the assessment of potential treatment resistance in schizophrenia.

**Significant outcomes**

- A significant number of patients thought to have treatment‐resistant schizophrenia have subtherapeutic antipsychotic plasma levels.
- Antipsychotic plasma levels may be of benefit in the assessment of treatment resistance in schizophrenia.

**Limitations**

- It is not possible to definitively determine whether the subtherapeutic plasma levels observed were due to non‐adherence as opposed to, for example, pharmacokinetic factors.
- There are inconsistencies in the relationships between plasma level and clinical response, and it may be that some patients classified as having a therapeutic level are undertreated.

## Introduction

Treatment‐resistant schizophrenia is defined as inadequate response to two or more adequate antipsychotic treatment episodes 1. It is a major challenge in the clinical management of schizophrenia, affecting about one in three patients 2, 3. Treatment‐resistant schizophrenia accounts for a disproportionate burden, both in terms of morbidity and healthcare costs 4. Neuroimaging studies have demonstrated that treatment resistance is not secondary to insufficient D2/3 receptor blockade 5, 6. Indeed, it is a requirement for all clinical definitions of resistant schizophrenia that individuals have received adequate treatment 1. What ‘adequate’ entails varies between clinical guidelines, but guidelines are consistent that lack of response must be secondary to medication ineffectiveness, not medication under exposure 1.

In an earlier pilot study 7, we found that 44% of individuals judged by their treating team to have treatment‐resistant schizophrenia had either subtherapeutic (25%) or undetectable (19%) antipsychotic plasma levels. Interindividual pharmacokinetic variation may lead to disparities in plasma levels following similar doses in different individuals. Intraindividually, however, a linear relationship exists between dose and plasma levels. As a result, therapeutic drug monitoring is not routinely recommended when prescribing non‐clozapine antipsychotics, as dose can be predictably titrated against clinical effect 8. The results of our initial pilot study suggested, however, that there may be a specific role for plasma level testing in the assessment of treatment resistance. In this study, we aimed to test this in a large patient sample, in a pragmatic cross‐sectional study of standard clinical practice. As secondary objectives, we also aimed to identify clinicodemographic factors associated with subtherapeutic/undetectable levels and to prospectively investigate the association between subtherapeutic/undetectable levels and subsequent hospitalization.

## Material and methods

### Participants

All individuals referred to the TREAT service, a community mental health service for the assessment and management of treatment‐resistant schizophrenia 9, between 1 January 2012 and 1 April 2017 were considered for inclusion in the study. This service covers all the community mental health teams in the London boroughs of Lambeth and Southwark (total population 641,100) 10. Patients considered treatment‐resistant are referred to the service as part of the clinical care pathway for patients with psychosis.

Participants were included if (i) the referring clinician considered the patient treatment‐resistant, based on their clinical assessment and knowledge of the patient's treatment history; (ii) the participant was currently taking an oral non‐clozapine antipsychotic; and (iii) the participant consented to antipsychotic plasma level testing.

Exclusion criteria included current treatment with clozapine because this is used to treat treatment resistance, treatment with a long‐acting injectable antipsychotic because the treatment is administered by staff so adherence is known, or treatment with multiple antipsychotics because it is not clear what constitutes a potentially therapeutic level with combination treatment.

Data collection was performed retrospectively from electronic records following approval by the South London and Maudsley NHS foundation trust audit committee. Permission was granted for the analysis of data obtained as part of routine clinical practice. As the tests and data were collected as part of routine clinical practice, patients were not required to provide separate consent.

### Data collection

**Figure 1:**
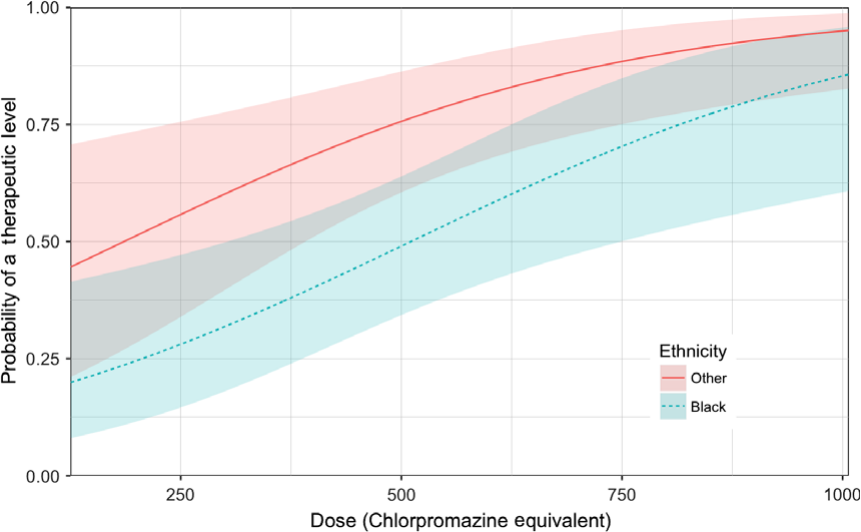
Antipsychotic dose (*P* = 0.01) and ethnicity (*P* = 0.02) were significant predictors of subtherapeutic plasma levels in a logistic regression (overall model *P* < 0.001).

Demographic details (age, sex and ethnicity), diagnosis, current antipsychotic, antipsychotic dose and length of treatment were obtained from clinical records (Fig. 1). Ethnicity was determined based on participant report, with ‘black ethnicity’ referring to individuals identifying as being of Black African or Black Caribbean descent. Dose was converted to chlorpromazine equivalents (CPZ) using ratios derived from consensus guidelines 11. Laboratory records for the year prior to assessment were examined to assess the frequency of plasma level monitoring in usual clinical practice.

Blood samples were taken at the time of first assessment by the TREAT service. Analysis methods and lower limits of quantification were as previously reported 7, 12. Plasma level testing is not recommended for standard dose adjustment. When assessing treatment resistance, however, a level below ranges associated with therapeutic response indicates potential under treatment 13. Levels were classified as therapeutic if they were above the following thresholds: amisulpride 200 μg/l 14, aripiprazole 150 μg/l 15, haloperidol 5 μg/l 16, olanzapine 20 μg/l 17, quetiapine 100 μg/l 8, risperidone 20 μg/l (including total risperidone and 9‐hydroxyrisperidone) 18 and sulpiride 200 μg/l 19. Levels below the threshold were classified as either subtherapeutic or undetectable.

Subsequent to the initial plasma level, participants’ case notes were reviewed up until 10 April 2017 to determine whether any in‐patient admissions occurred during this period.

### Statistical analysis

All statistical analysis was performed using r version 3.3.2 20. The ‘survival’ (version 2.41‐3) and ‘survivalroc’ (version 1.0.3) packages were used for survival analyses and the ‘boot’ (version1.3‐18) package used for bootstrapping confidence intervals. The ‘ggplot2’ (version 2.2.1) and ‘survminer’ (version 0.4.0) packages were used for figure construction. The normality of predictor variables was checked using the Shapiro–Wilks test. Differences between participants with therapeutic or subtherapeutic plasma levels, for normally distributed predictor variables, were determined using an independent samples *t*‐test. Differences between non‐normally distributed continuous variables were assessed with the Mann–Whitney *U*‐test. Pearson's chi‐squared test was used to test for group differences regarding categorical variables.

Logistic regression was then undertaken to determine the contribution of potential predictor variables to the outcome of interest (the binary outcome of presence/absence of a therapeutic plasma level). Predictor variables were included in this multivariate model if bivariate analysis had shown a significant association.

Kaplan–Meier survival analysis was used to determine if subtherapeutic plasma levels were associated with an increased risk of subsequent hospital admission. The Mantel–Cox log rank was used to test for significance, while Cox's regression model was used to calculate the hazard ratio. The assumption of proportional hazards was tested for by calculating Schoenfeld residuals, inspecting the relationship with time, and testing the significance of the time residual correlation coefficient. No assumptions regarding the linearity of independent variables were made given that a single categorical variable was used as a predictor. Time‐dependent sensitivity and specificity of a subtherapeutic plasma level's prediction of admission within 2 years was calculated 21, and basic bootstrap (5000 samples) estimated confidence intervals reported.

All tests of significance were two‐tailed, with a threshold of significance set at *P* < 0.05.

## Results

Plasma levels were obtained for 99 participants between 1 January 2012 and 1 April 2017. Initial findings for 33 of these participants have previously been reported 7. Demographic details of participants are shown in Table 1. Sixty‐four per cent of participants were male, 48% were of black ethnicity and the median age was 40.2 years (IQR 21.4).

**Table 1:** Demographic and clinical characteristics of study participants

	Therapeutic level	Subtherapeutic/undetectable	*P*
*N*, %	64 (65)	35 (35)	
Age in years, median (IQR)	44.4 (22.8)	35.7 (17.2)	0.04^a^
Male gender, *n* (%)	40 (63)	23 (66)	0.92^b^
Ethnicity, *n*			
White British/other	40	11	0.006^b^
Black	24	24	
Diagnosis, *n*			
Schizophrenia	47	23	0.47^b^
Schizoaffective	10	9	
Other	7	3	
Antipsychotic, *n*			
Amisulpride	11	6	0.11^b^
Aripiprazole	4	8	
Haloperidol	0	1	
Olanzapine	35	14	
Quetiapine	6	3	
Risperidone	6	3	
Sulpiride	4	0	
Length of treatment with current antipsychotic in months Median (IQR)	34.6 (403)	24.0 (192)	0.03^a^
Dose, CPZ equiv (11) mg/day Median, (IQR)	600 (585)	400 (55)	<0.001^a^
Mean level, % of minimum therapeutic range (SD)	531.7 (995)	30.3 (29.2)	
Admission, *n* (%)	7 (11)	11 (31)	0.02^c^

a Mann–Whitney.

b Pearson chi‐squared.

c Mantel–cox log rank.

Twelve per cent of participants had undetectable levels, and 23% had detectable but subtherapeutic levels. Further discussion of ‘subtherapeutic levels’ includes both groups – i.e. 35% of the total sample. Only two participants had had plasma levels measured in the year prior to their assessment. Forty‐nine per cent of participants were prescribed olanzapine, 12% aripiprazole, 17% amisulpride, 9% risperidone, 7% quetiapine, 4% sulpiride and 1% haloperidol. Median follow‐up time for the analysis of time to hospitalization was 1.46 years.

Clinicodemographic variables showed a non‐normal distribution. Bivariate testing demonstrated that younger age, black ethnicity, lower dose and shorter length of treatment were all significantly associated with subtherapeutic plasma levels (see Table 1). Gender, specific antipsychotic and symptom scores were not significantly associated with a subtherapeutic plasma level.

Antipsychotic dose, length of treatment and ethnicity were entered into a binomial logistic regression, with the presence of a therapeutic plasma level as the dependent variable. Age was not included in this analysis due to collinearity with length of treatment (*r*_p_ 0.44, *P* < 0.001). In the logistic regression, ethnicity and antipsychotic dose remained significant predictors, but length of treatment was no longer significant. A regression of solely antipsychotic dose and ethnicity showed that lower antipsychotic dose (odds ratio per 100 mg cpz equivalents = 1.43, 95% CI 1.14–1.81, *P* = 0.01) and black ethnicity (odds ratio 3.23, 95% CI 1.27–8.22, *P* = 0.02) predicted a subtherapeutic/undetectable plasma level (overall model *P* < 0.001; see Fig. 1). Individuals of black ethnicity were not prescribed antipsychotics at a significantly lower dose (*P* = 0.07).

**Figure 2:**
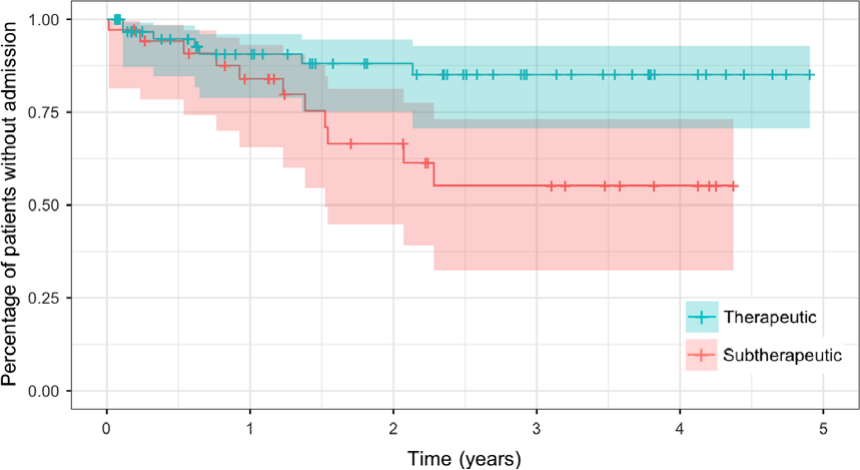
Time to hospitalization in participants with therapeutic (top line) or subtherapeutic (bottom line) antipsychotic plasma levels. Individuals with a subtherapeutic plasma level had a significantly higher risk of hospital admission (*P* = 0.019).

Rates of hospitalization were higher in individuals with a subtherapeutic plasma level (31%) compared to those with a therapeutic level (11%), and this was statistically significant (*P* = 0.019, hazard ratio 1.8, 95% CI 1.4–2.6; see Fig. 2). The assumption of proportional hazards was met in that no significant relationship between time and Schoenfeld residuals was observed (*P* = 0.16). A subtherapeutic plasma level at baseline had a sensitivity of 0.59 (95% CI 0.32–0.84) and a specificity of 0.70 (95% CI 0.59–0.78) to predict admission at 2 years.

## Discussion

To our knowledge, the current study is the largest evaluation to date of antipsychotic plasma levels in individuals with clinically defined treatment‐resistant schizophrenia. The main finding is that about one‐third of individuals considered by their treatment team to be treatment‐resistant had subtherapeutic or undetectable antipsychotic plasma levels. Individuals of black ethnicity and those prescribed lower antipsychotic doses were more likely to have a subtherapeutic/undetectable level. In addition, we found that individuals with a subtherapeutic/undetectable level had 80% greater odds of being subsequently admitted to hospital.

The present results are in keeping with the findings of our pilot study 7, and to our knowledge, there are no other studies examining plasma levels in the assessment of treatment resistance. A number of prospective studies have examined the relationship between plasma levels and clinical response 22, but these are typically prospective studies conducted in in‐patient settings, and address a separate question.

We are also unaware of any studies using non‐clozapine plasma levels to predict hospitalization. However, our results here are consistent with the well‐established finding that antipsychotic non‐adherence is associated with an increased risk of relapse 23, 24 and the finding that a high proportion of patients presenting with a psychotic relapse have subtherapeutic plasma levels 25.

Distinguishing treatment resistance from inadequate treatment is relevant to clinical practice, given that the management of each scenario differs. If a patient is treatment‐resistant, then clozapine treatment is the only licensed treatment with proven efficacy 26, 27, 28, 29. In contrast, where a patient has subtherapeutic or undetectable antipsychotic levels, assessment of adherence and other factors that may contribute to low antipsychotic levels is warranted, and the current findings support the suggestion that ideally a trial of a long‐acting injectable antipsychotic should be undertaken prior to diagnosing treatment resistance 1, 13.

The benefits of clozapine, however, appear to be primarily restricted to those resistant to non‐clozapine antipsychotics 30. Individuals with subtherapeutic plasma levels will likely be better served by addressing the causes of this rather than commencing clozapine treatment, particularly given the risks associated with sporadic adherence to clozapine 31. Given the high rates of non‐adherence to antipsychotic medication 32, it is likely that this may account for a substantial proportion of subtherapeutic/undetectable levels. Higher rates of non‐adherence to antipsychotics have previously been reported in people of black ethnicity 33, which may account for our finding of higher rates of subtherapeutic/undetectable levels in individuals of black ethnicity. Ethnicity, however, is an imprecise measure, encompassing a wide range of genetic and environmental factors. Furthermore, the grouping of individuals, from African and Caribbean descent, ignores potentially significant sociocultural differences between these two groups. As a result, it is not possible to determine which more specific underlying factor accounts for this finding. There are range potential causes other than non‐adherence that may also contribute to an individual's plasma level being subtherapeutic. Pharmacokinetic factors include rapid metabolism secondary to genetic variants affecting the function of metabolic enzymes 34, or enzyme induction secondary to smoking or other medications 35, 36. The association between lower antipsychotic dose and the presence of subtherapeutic levels is to be expected, and dose increases should be considered when other reasons for a low level have been excluded.

A subtherapeutic/undetectable plasma level was associated with subsequent hospital admission. This is consistent with the high rates of relapse observed following antipsychotic discontinuation 37. This suggests that individuals with a subtherapeutic level should be monitored particularly closely, and attempts made to promptly address the underlying reasons for the subtherapeutic level.

### Strengths and limitations

Strengths of the study included its naturalistic nature, and the fact that it included a large and representative group of patients. The initial assessment of treatment resistance by the patient's treating clinician was reflective of standard clinical practice, and as a result, our findings have relevance for clinical settings. While no control group of responsive patients was included, this would not have changed our primary finding, nor its interpretation.

The aim of the current study was to establish whether a significant proportion of patients believed to be treatment‐resistant were potentially in fact undertreated. Precisely, defining ‘under treatment’ is complicated by the fact that therapeutic ranges established for antipsychotics are approximate, and the evidence is limited in certain cases such as sulpiride and quetiapine 8. For most antipsychotics, the relationship between plasma level and response is not exact, and we therefore chose thresholds beneath which it was highly likely the individual was receiving insufficient antipsychotic exposure. As such, we were conservative in our lower boundary, and it is likely that several patients with ‘therapeutic levels’ may still have been undertreated. These individuals could therefore potentially benefit from increasing the dose of their current antipsychotic drug and determining response before considering clozapine treatment if response remains inadequate.

It is likely that a high proportion of subtherapeutic levels are secondary to limited adherence. The definitive assessment of adherence, however, is fraught with difficulty, with patient report, clinician judgment, pharmacy records and plasma levels all showing minimal agreement 32, 38. A limitation of the study is that without specifically testing pharmacokinetic factors in individual patients, it is not possible to rule these out and definitively attributes subtherapeutic plasma levels to low adherence. Another methodological limitation is that plasma levels were not taken at a set time following a participant's reported last dose. For the majority of antipsychotics, it is recommended that trough levels are measured, immediately prior to when the next dose is due 29. This is typically either late evening or early morning. However, these are inconvenient times for a patient to attend an out‐patient setting, making them impractical in clinical practice. The fact that samples were generally not earlier than trough, means that if participants were adherent their plasma levels would generally have been higher than their trough value. This means, if anything, we may have underestimated the prevalence of subtherapeutic levels and would not account for the presence of undetectable levels. Olanzapine is an exception to this, in that plasma sampling is recommended 12 h postdose 29. It is therefore possible that for some individuals prescribed olanzapine, a low level could merely reflect an extended length of time (up to 17 h) since their last dose. Of the 40 individuals taking olanzapine, 14 had subtherapeutic levels, and of these 11 had either undetectable levels or a level ≤10 μg/l. The half‐life of olanzapine ranges from 21 to 54 h; therefore, it is likely that for the majority of these subtherapeutic participants, even rapid metabolizers would have still had a subtherapeutic level if tested at 12 h postdose 31, 39. In addition, when individuals with olanzapine plasma levels of 10–20 μg/l were reclassified as having a therapeutic level the associations with dose, ethnicity and likelihood of hospitalization remained significant. In summary, while inconsistency in the timing of blood sampling could potentially have some effect on the magnitude of our results, it would not be expected to lead to differences in their statistical significance, interpretation or clinical relevance.

While plasma levels of several antipsychotics have been shown to directly correlate with D2/3 receptor occupancy in the brain, the correlation is not 100% 14, 40. Thus, it is also possible that a proportion of individuals with therapeutic plasma levels of antipsychotic may not have adequate D2/3 occupancy in the brain. It is also possible that some patients with no/subtherapeutic drug levels had received adequate treatment trials in the past with limited response. Given the difficulties in accurate retrospective assessment of response, however, it is recommended that treatment resistance not be diagnosed solely on the basis of retrospective report 1.

### Implications

Our finding that over one‐third of individuals clinically identified as treatment‐resistant have a subtherapeutic/undetectable plasma level has implications for clinical practice and trials of treatment resistance. It indicates that standard clinician evaluation that patients are receiving adequate antipsychotic treatment may be unreliable in one in three patients and supports the recent consensus recommendation for antipsychotic level testing in the assessment of patients for clinical studies of treatment‐resistant schizophrenia 1. While antipsychotic plasma levels should not be the sole factor in guiding clinical management, they may be of assistance, particularly in cases where response has been inadequate. While our study was unable to assess individual reasons for low levels, in clinical practice, this can generally be accomplished via careful clinical review, and appropriate action be undertaken in conjunction with the patient 13.

Guidelines recommend the use of plasma level testing not only in evaluating treatment resistance, but also in case of adverse effects and suspected non‐adherence. Despite this, it appears plasma level testing is rarely used in clinical practice 41, and in our sample, only two individuals had had plasma levels tested in the year prior to referral. Barriers to antipsychotic plasma level testing include a lack of availability, patient reluctance, and cost. The development of more accessible and acceptable methods for the monitoring of plasma levels may help with regard to the first two of these obstacles 42, 43. Costs will vary depending on location. At the Toxicology Unit, Department of Clinical Biochemistry, King's College Hospital, London, UK, plasma level testing is approximately £35 per sample. This expense must be viewed in proportion to both the cost of antipsychotic treatment (which can be around £90 per month for new antipsychotics 44), and the health and economic cost of potentially inappropriate pharmacological management.

In summary, a clinically significant proportion of individuals with schizophrenia thought to be treatment‐resistant have subtherapeutic antipsychotic plasma levels, secondary to non‐adherence or pharmacokinetic factors. This is more common in individuals of black ethnicity and those prescribed lower doses and is associated with an increased risk of subsequent hospitalization. The use of plasma level testing in the evaluation of treatment resistance has the potential to assist in optimizing individual clinical management.

## Declaration of interest

RM, KB, ED, JD, CG, SJ, SK, TP and MR declare no conflicts of interest. TRM has received honoraria as a speaker for Lundbeck and Pfizer. ODH has received investigator‐initiated research funding from and/or participated in advisory/speaker meetings organized by Astra‐Zeneca, Autifony, BMS, Eli Lilly, Heptares, Jansenn, Lundbeck, Lyden‐Delta, Otsuka, Servier, Sunovion, Rand and Roche. Neither Dr Howes nor his family has been employed by or has holdings/a financial stake in any biomedical company.
